# Cognitive flexibility in adolescence: Neural and behavioral mechanisms of reward prediction error processing in adaptive decision making during development

**DOI:** 10.1016/j.neuroimage.2014.09.018

**Published:** 2015-01-01

**Authors:** Tobias U. Hauser, Reto Iannaccone, Susanne Walitza, Daniel Brandeis, Silvia Brem

**Affiliations:** a University Clinics for Child and Adolescent Psychiatry (UCCAP), University of Zurich, Neumünsterallee 9, 8032 Zürich, Switzerland; b Wellcome Trust Centre for NeuroImaging, Institute of Neurology, University College London, United Kingdom; c PhD Program in Integrative Molecular Medicine, University of Zurich, Zurich, Switzerland; d Zurich Center for Integrative Human Physiology, University of Zurich, Zurich, Switzerland; e Neuroscience Center Zurich, University and ETH Zurich, Switzerland; f Department of Child and Adolescent Psychiatry and Psychotherapy, Central Institute of Mental Health, Medical Faculty Mannheim/Heidelberg University, 68159 Mannheim, Germany

**Keywords:** Adolescence, Development, Cognitive flexibility, Functional magnetic resonance imaging (fMRI), Reward prediction errors

## Abstract

Adolescence is associated with quickly changing environmental demands which require excellent adaptive skills and high cognitive flexibility. Feedback-guided adaptive learning and cognitive flexibility are driven by reward prediction error (RPE) signals, which indicate the accuracy of expectations and can be estimated using computational models. Despite the importance of cognitive flexibility during adolescence, only little is known about how RPE processing in cognitive flexibility deviates between adolescence and adulthood.

In this study, we investigated the developmental aspects of cognitive flexibility by means of computational models and functional magnetic resonance imaging (fMRI). We compared the neural and behavioral correlates of cognitive flexibility in healthy adolescents (12–16 years) to adults performing a probabilistic reversal learning task. Using a modified risk-sensitive reinforcement learning model, we found that adolescents learned faster from negative RPEs than adults. The fMRI analysis revealed that within the RPE network, the adolescents had a significantly altered RPE-response in the anterior insula. This effect seemed to be mainly driven by increased responses to negative prediction errors.

In summary, our findings indicate that decision making in adolescence goes beyond merely increased reward-seeking behavior and provides a developmental perspective to the behavioral and neural mechanisms underlying cognitive flexibility in the context of reinforcement learning.

## Highlights

• Adolescents and adults show differences in processing RPEs.
• Adolescents learn faster from negative prediction errors.
• The anterior insula activation may cause altered sensitivity to RPEs.

## Introduction

Adolescence is a time when many things in life change at a very high pace. Its start is marked by the onset of puberty, when fundamental physiological alterations take place (Blakemore et al., 2010). At the same time, peer relationships change markedly (Brown, 2004; Somerville, 2013) and it becomes often more important to please peers than to obey the parents. With the transition into higher education and professional career, also the demands in these domains change fundamentally. All of these changes demand to flexibly adjust to the new requirements, to disengage from previous and to engage in novel targets. Failure to adjust may cause social exclusion, dropout from school or even psychiatric disorders and it is therefore very important for adolescents to possess high cognitive flexibility (Crone and Dahl, 2012).

The reinforcement learning (RL) theory (Sutton and Barto, 1998) suggests that cognitive flexibility and adaptive learning are driven by reward prediction error (RPE) signals. These RPE signals indicate expectation violations. It is well established that RPE-like signals are encoded by dopaminergic midbrain neurons (Schultz, 2002; Schultz et al., 1997). For events which are better than expected, a positive RPE will be elicited which reflects a phasic increase in dopaminergic firing. For negative RPEs – encoding events that are worse than expected – a decrease in dopaminergic activity is found. Such RPE signals are projected to a decision making network including striatal, prefrontal, and insular regions (e.g., Blakemore and Robbins, 2012; Bromberg-Martin et al., 2010; Gläscher et al., 2009). Importantly, the RL theory provides a mechanistic view on the processes involved in cognitive flexibility and therefore enables us, at least partly, to overcome the merely descriptive level of behavioral analysis.

In cognitive neuroscience and neuropsychology, cognitive flexibility has mainly been operationalized by sudden and implicit shifts in reward contingencies that have to be detected based on external feedback (Scott, 1962). To test cognitive flexibility, probabilistic reversal learning tasks have often been used (e.g., Adleman et al., 2011; Britton et al., 2010; Clarke et al., 2004; Klanker et al., 2013; van der Plasse and Feenstra, 2008; Xue et al., 2013). In these tasks, the reward probabilities of the objects change unpredictably and the subjects have to learn these changes based on the feedback they receive. Computationally, these feedback-driven learning processes can well be described by using a RPE-learning model because the subjects learn entirely based on the feedback (e.g., Gläscher et al., 2009; Hauser et al., 2014a). The neural correlates of RPEs in probabilistic reversal learning tasks have been successfully examined in previous studies on healthy adults and found to positively correlate with mainly striatal and ventromedial prefrontal areas, and to negatively correlate with areas such as the dorsomedial prefrontal cortex and the anterior insula (Gläscher et al., 2009; Hampton et al., 2006; Hauser et al., 2014a, b).

So far, only little is known about the developmental trajectories of RPE processing. Despite the importance of RPE processing in adolescence, only few studies have investigated RPE processing in adolescents (Christakou et al., 2011; Cohen et al., 2010; Javadi et al., 2014a; van den Bos et al., 2012). While Cohen et al. (2010) found differential activations between adolescents and adults in striatal areas, van den Bos et al. (2012) were not able to replicate that finding, but found differences in the connectivity between the ventromedial prefrontal cortex and the ventral striatum. These studies, however, used learning tasks which did not include reversals and therefore investigated merely associative learning, but not cognitive flexibility.

In this study, we were interested to study RPE processing in the context of cognitive flexibility and therefore compared performance of healthy adolescents (12–16 years) to adults using a probabilistic reversal learning task. By using a modified RL model, we compared the learning mechanisms during adaptive learning. Furthermore, we investigated RPE processing differences using functional magnetic resonance imaging (fMRI). Because previous studies found neural changes in activity in striatal and medial prefrontal areas (Christakou et al., 2011; Cohen et al., 2010; van den Bos et al., 2012), we hypothesized that these areas might also show altered RPE signals in the context of cognitive flexibility. Additionally, we hypothesized the anterior insular activity to be altered, because this region is crucially involved in RPE processing (Pessiglione et al., 2006; Seymour et al., 2004; Voon et al., 2010; Wittmann et al., 2008), it is highly relevant for error processing (Dosenbach et al., 2006) and it is known to show specific activation patterns during adolescence (Smith et al., 2014).

## Materials and methods

### Participants

Thirty-seven subjects participated in this study. One participant (13.0 y, m) had to be excluded prior to analysis due to excessive movement (> 2.5 mm scan-to-scan motion). The adolescent group consisted of 19 participants between 12 and 16 years (14.7 y ± 1.3, 10 females). The adult group consisted of 17 participants between 20 and 29 years (25.6 y ± 2.4, 10 females). All participants were right-handed and none reported any neurologic or psychiatric disorder. During scanning, there was no difference in movement between both groups (scan-to-scan movement: adults: mean = .079 mm ± .021; adolescents: mean = .076 mm ± .016; *t*(34) = .496, *p* = .623). Data from 15 adults (Hauser et al., 2014a) and all adolescents (Hauser et al., 2014b) were already used in previous articles. The study was approved by the local ethics committee and all adult participants gave written informed consent. For the adolescent group, the participants and their parents signed the consent form.

### Task

**Fig. 1:**
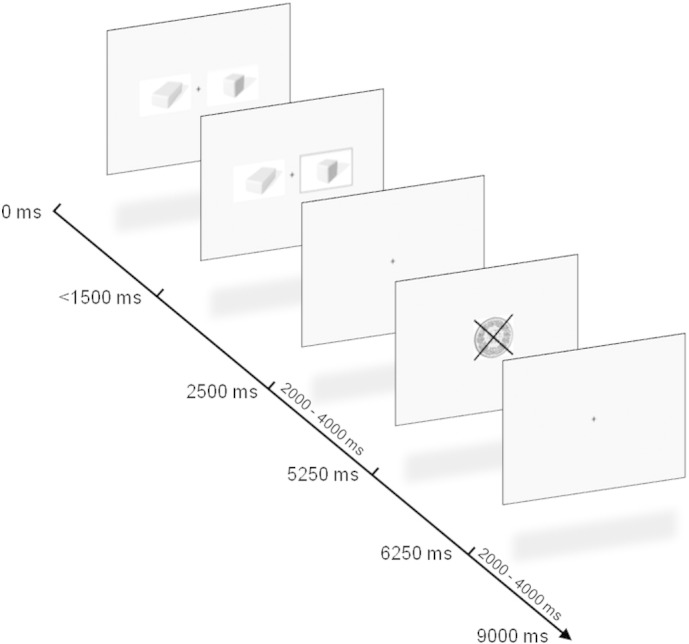
Probabilistic reversal learning task. On each trial (average duration: 9000 ms), two stimuli were simultaneously presented. The participant had to select one of the stimuli within 1500 ms. The selected stimulus was highlighted until the end of the stimulus presentation (2500 ms). After a jittered interstimulus interval (2000–4000 ms), the outcome was displayed for 1000 ms. Rewards were indicated by a framed coin whereas punishments were depicted by a crossed coin. Between trials, a jittered fixation cross was shown (2000–4000 ms).

The participants performed a probabilistic reversal learning task (Fig. 1; cf. Hauser et al., 2014a) while functional magnetic resonance imaging (fMRI) was recorded. The participants had to learn on a trial-and-error basis which of two presented stimuli was associated with the higher reward probability. One of the two stimuli was determined to be the correct stimulus and was rewarded with probability of 80%. The other stimulus was assigned with a reward probability of 20% and was punished in 80% of the trials. After the subject made at least 6 correct choices (maximum of 10 correct choices, randomly determined), a reversal of the reward probabilities occurred. Of the correct choices, at least 3 choices had to be consecutively correct to ensure that the subjects learned the association properly. When a reversal occurred, the previously correct stimulus became the incorrect stimulus, and vice versa. The possibility of reversals occurring was communicated to the participants beforehand, but they were not provided with any details about the frequency of the reversals. As a reward, the participants received 50 Swiss Centimes (approx. $0.50), whereas punishments resulted in a loss of 50 Swiss Centimes. The participants performed two runs of 60 trials each. Additionally, 20 null trials (9000 ms length) were randomly presented in each run. To force the participants to minimize misses, late answers were punished by subtracting 100 Swiss Centimes.

### Reinforcement learning models

We compared three different reinforcement learning models. Besides a standard Rescorla–Wagner model (Rescorla and Wagner, 1972), we implemented a model which had different learning rates for positive and negative RPEs. A similar model has already been used in adolescent decision making (van den Bos et al., 2012) and was implied to be more risk-sensitive (cf. Niv et al., 2012). Given that we have previously shown that reinforcement learning models with anticorrelated valuation fitted this task better than a standard Rescorla–Wagner model (Hauser et al., 2014a), we evaluated the risk-sensitive model with an anticorrelated valuation (RSAV) extension as a third model.

#### Rescorla–Wagner model

RPEs were computed as the difference between the expected (*V*_*t*_^*Chosen*^) and the received (*R_t_*) outcome at each trial t.

(1) $$RPE_{t}=R_{t}-V_{t}^{\mathrm{Chosen}}$$

The value of the chosen object was updated using the RPE, whereas the value of the unchosen object (*V*_*t* + 1_^*Unchosen*^) did not change its value.

(2) $$V_{t+1}^{\mathrm{Chosen}}=V_{t}^{\mathrm{Chosen}}+\alpha RPE_{t}$$

(3) $$V_{t+1}^{\mathrm{Unchosen}}=V_{t}^{\mathrm{Unchosen}}$$

where α is the learning rate.

#### Risk-sensitive model

In a seminal paper by Niv et al. (2012), the authors showed that tasks, where risk or outcome variance is not explicitly available, individual risk sensitivity can be assessed by using different learning rates for positive and negative RPEs. The chosen value *V*_t+1_^Chosen^ was therefore updated depending on the sign of the RPE

(4) $$V_{t+1}^{\mathrm{Chosen}}=V_{t}^{\mathrm{Chosen}}+\alpha^{+/-}RPE_{t}$$

whereas the value of the unchosen object was not changed Eq. (3). For positive RPEs, chosen values were updated using the free parameter *α^+^*, whereas for negative RPE, *α*^−^ was defined as the learning rate.

#### Risk-sensitive model with anticorrelated valuation (RSAV)

In reversal learning tasks, the feedback about the chosen object also informs about the value of the unchosen stimulus. Therefore, we and others used RPEs also to update the unchosen option (Gläscher et al., 2009; Hauser et al., 2014a). Here, we implemented the anticorrelation in the risk-sensitive model:

(5) $$V_{t+1}^{\mathrm{Chosen}}=V_{t}^{\mathrm{Chosen}}+\alpha_{\mathrm{Chosen}}^{+/-}RPE_{t}$$

(6) $$V_{t+1}^{\mathrm{Unchosen}}=V_{t}^{\mathrm{Unchosen}}-\alpha_{\mathrm{Unchosen}}^{+/-}RPE_{t}$$

where *α*_*Chosen*/*Unchosen*_^+/−^ describes the free parameter, which is different for chosen and unchosen options and for positive and negative RPEs.

To derive the action probabilities, we used a softmax action selection function in all models:

(7) $$p\left(A_{t}\right)=\frac{1}{1+e^{-\left(V_{t}^{A}-V_{t}^{B}\right)\tau }}$$

(8) $$p\left(B_{t}\right)=1-p\left(A_{t}\right)$$

where *V*_*t*_^*A*^ denotes the value of object *A* at time *t* and *τ* denotes a free parameter.

### Model estimation and comparison

For each participant, we estimated the maximum log-likelihood (cf. Hampton et al., 2006) using a genetic search algorithm (Goldberg, 1989) in Matlab, similar as in our previous study (Hauser et al., 2014a):

(9) $$\log L=\frac{\sum B_{\mathrm{switch}}\log P_{\mathrm{switch}}}{N_{\mathrm{switch}}}+\frac{\sum B_{\mathrm{stay}}\log P_{\mathrm{stay}}}{N_{\mathrm{stay}}}$$

The behavioral component B indicates whether the participant switched on the subsequent trial and *P* indicates the estimated probability to switch or stay.

Akaike information criterion (AIC; Akaike, 1973) was used to compare the models (cf. Hampton et al., 2006):

(10) $$AIC=-2\log L+2\frac{M}{N}$$

where *M* describes the number of free parameters and *N* is the number of trials. To choose the best-fitting among all models, we used Bayesian model selection for groups (Stephan et al., 2009).

In order to investigate whether the groups differed in their learning mechanisms, we fitted the free parameters of the best fitting model (RSAV model) to the behavior of each participant. These individual parameter estimates were subsequently compared between the age-groups.

For the fMRI analysis, we estimated one single set of canonical model parameters (*α*_*c*_^+^, *α*_*c*_^−^, *α*_*u*_^+^, *α*_*u*_^−^, τ) for all participants, similarly as in previous studies (e.g., Pine et al., 2010; Seymour et al., 2012; Voon et al., 2010). We decided to do so, because we were not interested to model any behavioral differences into our fMRI regression analysis and in order to obtain canonical and stable parameter estimates.

### Data acquisition

fMRI was conducted using a 3T Achieva (Philips Medical Systems, Best, the Netherlands), equipped with a 32-element receive head coil array. The echo-planar imaging (EPI) sequence was designed to minimize susceptibility-induced signal dropouts in orbitofrontal regions (40 slices, 2.5 × 2.5 × 2.5 mm voxels, 0.7 mm gap, FA: 85° FOV: 240 × 240 × 127 mm, TR: 1850 ms, TE: 20 ms, 15° tilted downward of AC-PC). Additionally, we simultaneously recorded 64-channel EEG and two electrocardiogram (ECG) channels using MR-compatible amplifiers (BrainProducts GmbH, Gilching, Germany). ECG signals were used to minimize cardioballistic artifacts in the fMRI data (see below). The present article focusses on the presentation of the fMRI data.

### fMRI data analysis

fMRI analysis was conducted using SPM8 (http://www.fil.ion.ucl.ac.uk/spm/). The EPIs were realigned and coregistered to the T1 image. Normalization was performed using the deformation fields which were generated using new segmentation. This resulted in a standard voxel size of 1.5 mm. Finally, spatial smoothing (6 mm full width at half maximum kernel) was conducted.

For the main effect analysis of RPEs in cognitive flexibility, we entered the model-derived RPEs as parametric modulator at the time of feedback into the first-level analysis. We additionally entered several regressors-of-no-interest into the GLM to improve model validity: choice values (value of chosen object) as parametric modulator at cue presentation, realignment-derived movement parameters, scan-to-scan movements greater than 1 mm, and cardiac pulsations (http://www.translationalneuromodeling.org/tapas/; Glover et al., 2000; Kasper et al., 2009). Furthermore, we regressed out missing answers and the temporal and spatial derivatives of all task-related regressors.

To analyze the main effect of RPEs at the second level, we entered all participants in a common random-effects analysis. The significance threshold was set to *p* < .05 voxel-height family-wise error (FWE) correction. For a better understanding of the RPE effects in each group, we displayed the RPE effects for each group separately in the supplementary material (Figs. S1, S2).

To obtain differential activations between our age groups, we restricted our analysis to areas which were involved in RPE processing (mask of whole-group effect at level *p* < .05 FWE, cf. Table 2) and carried out independent-sample t-tests. For the group comparison at second level, a significance threshold of *p* < 0.05 cluster-extent FWE was used (voxel height threshold *p* < .001). An unrestricted whole-brain group comparison is shown in the supplementary Fig. S3.

To better understand how the group differences are caused, we conducted a second, exploratory analysis of the functional differences in the areas which were significant in the group comparison using rfxplot (Gläscher, 2009). To do so, we conducted a post-hoc analysis of the significantly different cluster (here: aIns) and split the RPEs into three equally sized bins: negative, neutral (boundaries: adolescents: [− 0.30 ± 0.23, 0.18 ± 0.04], adults: [− 0.25 ± 0.18, 0.19 ± 0.05]) and positive RPEs. The boundaries did not differ between the groups (lower: *t*(34) = .64, *p* = .527; upper: *t*(34) = .70, *p* = .490). We compared the neural responses in these bins using repeated measures ANOVAs and post-hoc *t*-tests, corrected for multiple comparisons using Bonferroni correction.

## Results

### Behavior

Both groups performed the task equally well with 73.83% (± 4.4%) correct responses in adults and 73.38% (± 4.6%) in adolescents (*t*(34) = .296, *p* = .769). The groups also did not differ in the number of reversals which they performed. The adults switched on average 23.35 (± 8.80) times and the adolescents reversed 26.11 (± 8.31) times (*t*(34) = − .965, *p* = .341). Interestingly, we found a marginally decreased number of punishments before the adolescents switched (*t*(34) = 1.71, *p* = .097, adolescents: 1.56 ± 0.22, adults: 1.71 ± 0.30).

### Model comparison and parameters

**Fig. 2:**
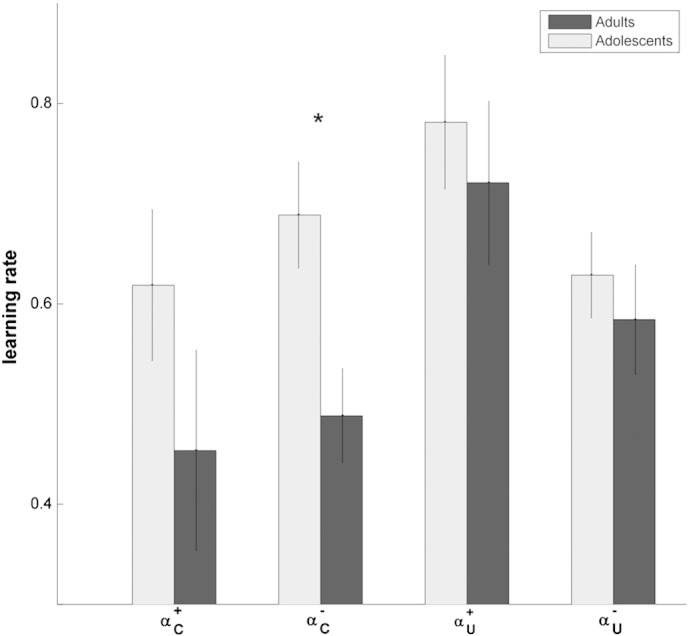
Learning rate differences between adolescents and adults. The parameters from the RSAV model show an increased learning rate for negative RPEs in chosen stimuli (*α*_*c*_^−^). The other learning rates did not significantly differ. *: *p* < .05, multiple comparison corrected.

The RSAV model clearly outperformed the other models across all subjects, as well as in both groups separately (Table 1). To evaluate whether model parameters were different between the groups, we conducted a repeated measures ANOVA with between-subject factor group (adults, adolescents) and within-subject factor parameter (*α*_*c*_^+^, *α*_*c*_^−^, *α*_*u*_^+^, *α*_*u*_^−^, *τ*). We found a significant difference between the free parameters (F_(4,136)_ = 73.45, *p* < .001) as well as an interaction between the parameters and the group (F_(4,136)_ = 2.851, *p* = .026). Post-hoc *t*-tests revealed that adolescents had a significantly increased learning rate for negative RPEs in chosen objects (*α*_*c*_^−^: adults: .49 ± .05, adolescents: .69 ± .05, *t*(34) = − 2.816, *p* = .04, multiple comparison corrected, Fig. 2), whereas the other parameters did not differ significantly (*α*_*c*_^+^: adults: .45 ± .10, adolescents: .62 ± .07, *t*(34) = − 1.336, *p* = .95; *α*_*u*_^+^: adults: .72 ± .08, adolescents: .78 ± .07, *t*(34) = − .581, *p* = 1.00; *α*_*u*_^−^: adults: .58 ± .06, adolescents: .63 ± .04, *t*(34) = − .636, *p* = 1.00; τ: 2.4 ± .2, adolescents: 1.9 ± .2, *t*(34) = 1.595, *p* = .60).

### fMRI analysis

#### RPE in cognitive flexibility

**Fig. 3:**
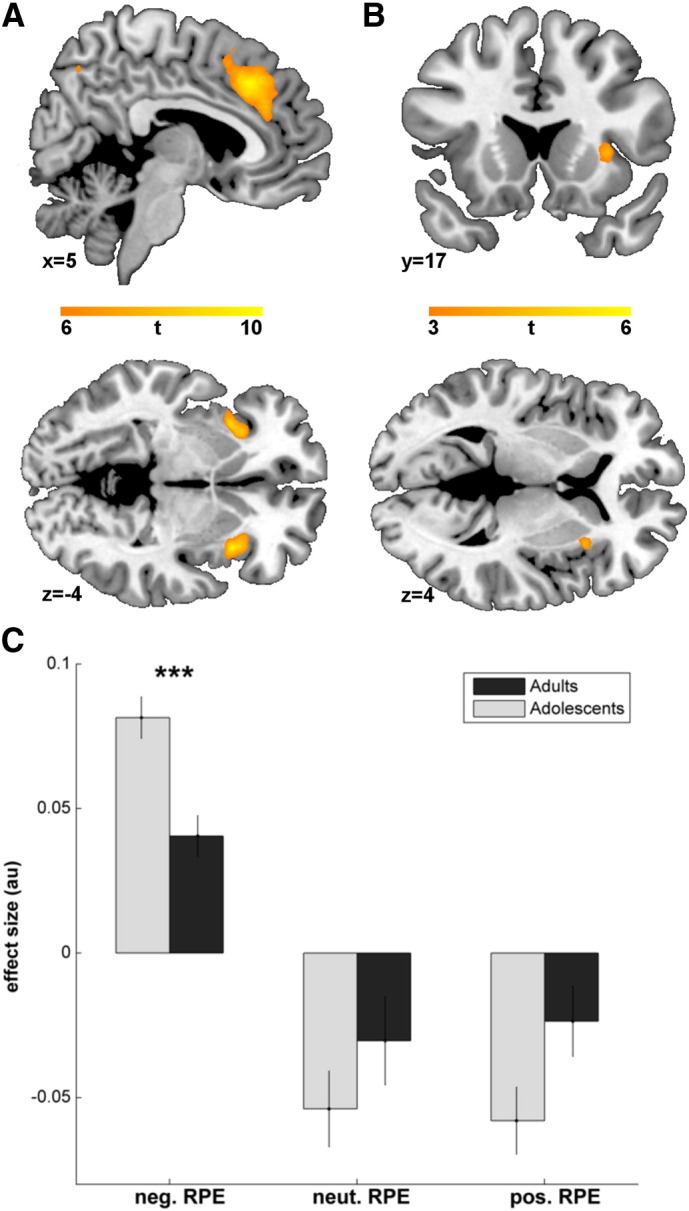
Differences between adolescents and adults in the RPE network. (A) A network containing the dmPFC (upper panel) and the aIns (lower panel) shows increased activation for decreasing RPEs among all subjects. (B) A group comparison between the adolescents and adults reveals a significant activation difference in the right aIns. (C) Subsequent exploratory analysis revealed that this group difference was mainly driven by an increased activation for negative RPEs in adolescents. ***: *p* < .001.

In our main effect analysis of RPEs in cognitive flexibility, we found areas which are typically positively correlated with RPEs (increasing RPEs elicit more activity) such as the putamen, ventromedial prefrontal cortex (vmPFC), amygdala and the posterior cingulate (Table 2). The bilateral anterior insula (aIns), bilateral dorsomedial prefrontal cortex (dmPFC), and the dorsolateral prefrontal cortex were significantly anticorrelated with RPEs (decreasing RPEs elicit more activity, Table 2, Fig. 3A).

#### Group comparison

We analyzed whether the responses within the RPE network significantly differed between the groups. We found one significant cluster in the right aIns (peak MNI *x* = 33, *y* = 18, *z* = 3; *t* = 4.60, *k* = 33, Fig. 3B) which showed increased activation for decreasing RPEs in adolescents. We did not find any significantly increased activation for positive RPEs in adolescents.

To better understand how the aIns differed in activation between adolescents, we decided to conduct an exploratory analysis of this cluster. We divided the RPEs in three equally sized bins of positive, neutral and negative RPEs. The repeated measures ANOVA with factors group (adolescents, adults) and RPE (negative, neutral, positive) revealed a significant RPE-effect (indicating that the aIns is modulated by RPEs across all subjects, F_(2,34)_ = 40.37, *p* < .001), a significant group-by-RPE interaction (indicating that only some RPE bins differ between groups, F_(2,34)_ = 4.60, *p* = .013), but no significant group effect (indicating that the group difference was not caused by generally increased or decreased responses across all RPE bins, F_(1,34)_ = 1.77, *p* = .193). Post-hoc *t*-tests of the three bins revealed that the interaction was caused by a significantly increased response to negative RPEs in adolescents compared to adults (*t*(34) = − 4.08, *p* < .001, corrected for multiple comparisons, Fig. 3C). Neutral (*t*(34) = 1.18, *p* = .734) and positive RPEs (*t*(34) = 2.06, *p* = .142) were not significantly different. This suggests that the difference in the aIns was mainly driven by the most negative RPEs, which is well in line with our behavioral finding of the increased learning rate for negative RPEs.

## Discussion

In this study, we investigated developmental aspects of cognitive flexibility using the mechanistic learning and decision making framework of reinforcement learning theory. By using an advanced reinforcement learning model, we found that adolescents learn more quickly from negative RPEs than adults. This implies that adolescents adjust their behavior more quickly after feedbacks which are worse than they expected.

Interestingly, most previous studies which investigated cognitive flexibility found strong performance improvements during childhood, but less behavioral differences between adolescents and adults (e.g., Crone et al., 2004, 2008; Hämmerer et al., 2011; Welsh et al., 1991; Wendelken et al., 2012). When looking at the behavior in our groups without using computational models, we do not find any difference in overall task performance or the number of switches, similar to the findings by Hämmerer et al. (2011). The marginally significant difference in the number of punishments before switches, however, points to the increased learning rate that we found in our modeling approach. This suggests that the use of reinforcement learning methods to study cognitive flexibility may be more sensitive to differences in the learning process than common behavioral analyses.

Previous studies on adolescent decision making under uncertainty found that adolescents are reward driven and behave rather risk seeking (e.g., Figner et al., 2009; Tymula et al., 2012). Therefore, our finding that adolescents are more sensitive to negative RPEs might appear to be somewhat contradictory on the first sight. However, we do not think that these results are conflicting, because these studies that found increased reward seeking did not investigate cognitive flexibility. Usually, tasks which were used to study reward seeking had different reinforcement structures which did not involve sudden changes in reward contingencies. Namely, these tasks often merely required to learn the association between a stimulus and a (probabilistic) outcome (e.g., Cohen et al., 2010; van den Bos et al., 2012). They did not require to detect environmental changes and to continuously adjust to changes in the reward contingencies. Therefore, negative RPEs have decreasing impact for the subjects' learning process over the course of the task: The negative RPEs carry information about the value of stimuli (similarly as positive RPEs), but they do not indicate changes in reward structures. In our task, however, negative RPEs continue to be essential, given that they carry important information about changes in reward contingencies. We therefore think that the increased sensitivity which we found in this study may reflect an additional aspect to differ between adolescent and adult decision making, apart from the reward seeking behavior in adolescents in tasks unrelated to cognitive flexibility.

In our fMRI analysis of RPEs, we replicated previous studies showing that RPEs are positively associated with a decision making network containing the striatum and vmPFC (e.g., Gläscher et al., 2009; Rutledge et al., 2010; Voon et al., 2010; Table 2), in which both areas are associated with valuation, value comparison and evaluation of objects (e.g., Gläscher et al., 2010; Hunt et al., 2013). Additionally, the RPEs anticorrelated with dmPFC and the aIns (Fig. 3A, Table 2), meaning that activity in this area increases with decreasing RPEs. These areas are important hubs for cognitive control and affective processing and are thought to guide behavioral adaptation (Cavanagh and Frank, 2014; Critchley, 2005; Hampton and O'Doherty, 2007).

In the group comparison, we found a significantly different activation in the aIns. No difference was found in the other areas of the RPE network. The neural responses of the aIns support our behavioral finding that adolescents were more sensitive to negative RPEs: the differential activation was mainly driven by the most negative RPEs, while neutral or positive RPEs did not elicit significantly different responses per se.

The aIns is a central hub in the brain and is one of the most commonly activated areas in human neuroimaging studies (Nelson et al., 2010). It is activated in a wide variety of cognitive and emotional tasks (Dosenbach et al., 2006) and forms the important salience network in resting state literature (Menon and Uddin, 2010). Unsurprisingly, the aIns has been ascribed to a wide variety of functions from processing visceral and emotional information (Critchley, 2005) to controlling attention and task demands (Dosenbach et al., 2006, 2007; Nelson et al., 2010). The aIns is also crucially involved in decision making and similar tasks. It has been found to process RPEs (Pessiglione et al., 2006; Seymour et al., 2004; Voon et al., 2010; Wittmann et al., 2008), it indicates (feedback) errors with a high reliability (Dosenbach et al., 2006), and it has a high predictive value for task switching in a similar cognitive flexibility task (Hampton and O'Doherty, 2007). This is also in line with the assumption that the aIns is involved when a feedback is processed consciously (Nelson et al., 2010; Wheeler et al., 2008). Moreover, the aIns has been associated with processing information about risk (Burke and Tobler, 2011; Ishii et al., 2012; Paulus et al., 2003; Preuschoff et al., 2008).

Differences in aIns activity have often been found in the developmental literature. Previous studies found developmental effects during tasks of cognitive flexibility (e.g., Christakou et al., 2009; Rubia et al., 2006; Smith et al., 2011) and in other cognitive domains (Christakou et al., 2011; Jarcho et al., 2012; Keulers et al., 2011; Masten et al., 2009; Somerville et al., 2011; Van Leijenhorst et al., 2010). However, the developmental importance of this area has largely been neglected.

Given the wealth of information about aIns functioning, one could speculate about how the increased activity in adolescents might be related to their increased learning rate. It is well known that aIns activity often coincides with activation in the dmPFC (cf. Hauser et al., 2014a, 2014b; Nelson et al., 2010; Seymour et al., 2004). However, it is assumed that the dmPFC is mainly involved in processing cognitive aspects, whereas the aIns rather processes visceral and emotional information (Nelson et al., 2010). The increased insular activity (esp. to negative RPEs) might indicate that adolescents weight the emotional information more strongly which then leads to a faster adaptation from negative feedbacks. This idea is in line with the assumption by Van Leijenhorst et al. (2010) who also found increased insular activity and associated it with increased physiological arousal. Additionally, it fits well with Crone and Dahl's suggestion that adolescence is a time when affective systems are a major driving force for goal selection and decision making (Crone and Dahl, 2012).

Lately, Smith et al. (2014) reviewed developmental studies with respect to the aIns and integrated them into a new neurodevelopmental theory of adolescent decision making. The authors state that the aIns – as being a cognitive-emotional hub – is immaturely connected during adolescence and therefore adolescents are biased toward affectively driven decisions. This notion seems to be well in line with Crone and Dahl's idea of a dominant social-affective system (Crone and Dahl, 2012), and also fits well with our findings in this study.

Very recently, Javadi et al. (2014a, b) published two papers from their study on developmental effects in decision making. Similarly to our study, the authors also used a probabilistic reinforcement learning task and used computational algorithms to infer their learning mechanisms. The authors (Javadi et al., 2014a) found an increased decision noise in their adolescent sample compared to healthy adults. However, the authors did not find any differences in prediction error processing in their regions-of-interest - despite their large adolescent sample. There are several crucial differences in their analysis which possibly are responsible for the diverging findings. In their behavioral modeling, Javadi et al. (2014a) used a Rescorla–Wagner model which does not differentiate between learning from positive and negative RPEs (Krugel et al., 2009; Rescorla and Wagner, 1972). Therefore, it is evident that the authors could not detect an increased learning rate for negative RPEs. Interestingly, the authors report a marginally different switching probability after correctly punished trials—similar as in our study. Additionally, their learning model seems to only update the chosen, but not the unchosen option. We and others previously demonstrated that models which update both options are better suited to model such a probabilistic reinforcement learning tasks (Gläscher et al., 2009; Hauser et al., 2014a). Moreover, in their fMRI analysis, the authors only analyzed responses in the anterior cingulate, ventral striatum and the vmPFC (Javadi et al., 2014a, b). Similar as in our study, they did not find any RPE differences in these areas. Unfortunately, the authors did not report any analysis of the aIns. Therefore, it cannot be determined whether their aIns showed similar developmental changes in RPE processing.

RPE-like signals are assumed to reflect a general neural update signal in a variety of domains, not only in decision making (Friston, 2010; Iglesias et al., 2013). Therefore, an increased insular sensitivity to negative RPEs might not only affect decision making, but also other areas in which adolescence reflects a unique period, such as in social interactions or psychiatric disorders. Adolescents are known to be more sensitive to the presence of peers (Chein et al., 2011) and peer rejection (Masten et al., 2009), and show a markedly increased prevalence in psychiatric disorders, such as anxiety, depression or substance abuse (Kessler et al., 2005, 2007). Although these problems are well known, it has only recently been suggested that they might have a common neural basis (Paus et al., 2008). The aIns seems to be crucial in all three domains. It is strongly involved in empathy-related processes (Singer et al., 2004, 2009) and social rejection (Masten et al., 2009). It has also been associated with depression, anxiety or substance abuse (for reviews cf. Craig, 2009; Naqvi and Bechara, 2009). Based on the idea that the aIns is an integrative hub which associates cognitive and affective–visceral information, one could speculate that overly strong (negative) prediction errors in the insular cortex reflect an overly dominant affective feedback. If an adolescent is not able to cognitively down-regulate such strong prediction errors (caused by social interactions, visceral inputs or homeostatic imbalances), she/he may use other strategies to suppress these signals. Such alternative strategies could entail to externally manipulate affective inputs (e.g., by taking neuroactive substances), or to adjust internal expectations and beliefs (e.g., catastrophic thinking in anxiety (Hofmann, 2005) or learned helplessness in depression (Seligman, 1992)). However, there is very little evidence for such mechanisms so far and further studies are urgently needed to investigate the extent to which activation differences in the aIns also play a role in adolescent social interactions or juvenile psychiatric disorders.

In this study, the age spectrum of our adolescent group had a relatively large age range (12–16 years). We sampled from a large age-width of the adolescence spectrum, because we wanted to draw conclusions which are generalizable for most of adolescence. If one only investigates a small age-range, it is unclear whether the differences are highly specific for only this age or whether they have validity for the whole period of adolescence. With our approach, however, we are not able to detect differences which may only occur early or late in adolescence. Additionally, given the relatively small sample size, we were also not able to look at age-related changes during adolescence. In further studies, it is essential to increase sample sizes and/or to use longitudinal designs to determine whether the learning trajectories (and their neural correlates) show changes also within the period of adolescence.

## Conclusions

Taken together, our findings expand the current knowledge of adolescents learning and decision making. While adolescents have often been described as reward-driven and risk-seeking (Blakemore and Robbins, 2012; Galvan, 2010), we were able to show that in the context of cognitive flexibility, adolescents are more sensitive to negative RPEs than adults. This novel finding suggests that decision making in adolescence goes beyond merely increased reward-seeking behavior—at least in the context of cognitive flexibility. Our neuroimaging results suggest that this difference is likely to be caused by an altered response of the aIns. It is well established that the aIns receives dopaminergic innervations (Gaspar et al., 1989) and processes dopamine-associated RPEs. Whether the altered response of the aIns is driven by similar changes in the dopaminergic system as suggested for reward seeking behaviors (Galvan, 2010; Spear, 2000; Steinberg, 2008) remains, however, unclear and should be examined in future studies.

## Figures and Tables

**Table 1:** Results of the model comparison. Model comparison clearly revealed that the RSAV model has a better model fit than the Rescorla–Wagner and the risk-sensitive model in both groups (mean ± SD). logL: maximum log-Likelihood, AIC: Akaike Information Criterion, p_x_: exceedance probability (probability that the given model fits data better than the other models).

Model	All subjects			Adolescents			Adults		
	logL	AIC	p_x_	logL	AIC	p_x_	logL	AIC	p_x_
Rescorla–Wagner	− 0.98 ± 0.12	1.999 ± 0.248	0	− 0.98 ± 0.14	1.997 ± 0.271	0	− 0.98 ± 0.11	2.001 ± 0.229	0
Risk–sensitive	− 0.97 ± 0.13	1.985 ± 0.250	0	− 0.97 ± 0.14	1.988 ± 0.282	0	− 0.97 ± 0.11	1.981 ± 0.219	0
RSAV	− 0.66 ± 0.21	1.407 ± 0.411	**1**	− 0.67 ± 0.23	1.424 ± 0.464	**1**	− 0.65 ± 0.18	1.387 ± 0.356	**1**

**Table 2:** Reward prediction errors in cognitive flexibility. Regions which correlate with RPEs across all subjects (p < .05 FWE; only clusters with k > 29 are listed). All coordinates are reported in MNI space. RPE: increasing activity with increasing RPEs; − RPE: decreasing RPEs elicit more activity; aIns: anterior insula; amygd: amygdala; dmPFC: dorsomedial prefrontal cortex; dlPFC: dorsolateral prefrontal cortex; IPL: inferior prefrontal cortex; mPFC: medial prefrontal cortex; PCC: posterior cingulate cortex; SFG: superior frontal gyrus; vmPFC: ventromedial prefrontal cortex.

Contrast	Region	Hemisphere	Cluster size (voxels)	*x*	*y*	*z*	*z* score
RPE	amygd	Right	95	18	− 7.5	− 18	6.74
		Left	69	− 27	− 9	− 19.5	6.03
	putamen	Left	99	− 27	− 13.5	1.5	6.40
	mPFC	Left	132	− 9	55.5	18	6.05
	IPL	Left	64	− 48	− 63	22.5	5.97
	SFG	Left	30	− 18	30	45	5.93
	PCC	Left	133	− 6	− 54	12	5.91
	precentral	Right	50	55.5	0	6	5.89
	vmPFC	Left	189	− 10.5	42	− 10.5	5.83
− RPE	dmPFC	Bilateral	1712	1.5	28.5	39	7.15
	aIns	Right	622	36	18	− 1.5	6.90
		Left	326	− 34.5	16.5	− 6	6.52
	dlPFC	Right	196	25.5	48	27	6.45
			163	39	31.5	33	5.82
	IPL	Right	112	55.5	− 42	43.5	6.24
			35	37.5	− 42	42	5.91
		Left	89	− 36	− 46.5	40.5	5.95
	Precuneus	Bilateral	65	7.5	− 66	48	6.14

## Appendix A Supplementary data

Supplementary information. **Figure S1**. RPE effects in adolescents. **Figure S2**. RPE effects in adults. **Figure S3.** Whole-brain differences between adolescents and adults in RPE processing.
