# Supplementary material for: Cognitive flexibility in adolescence: Neural and behavioral mechanisms of reward prediction error processing in adaptive decision making during development
Source: Neuroimage. 2015 Jan 1;104:347–54. doi: 10.1016/j.neuroimage.2014.09.018 (PMC4330550; doi:10.1016/j.neuroimage.2014.09.018)
Supplement: Supplementary file 1 — Supplementary information. Figure S1. RPE effects in adolescents. Figure S2. RPE effects in adults. Figure S3. Whole-brain differences between adolescents and adults in RPE processing. [file mmc1.doc]

**Cognitive flexibility in adolescence: Neural and behavioral mechanisms of reward prediction error processing in adaptive decision making during development**

Tobias U. Hauser, Reto Iannaccone, Susanne Walitza, Daniel Brandeis, & Silvia Brem

**Supplementary Material**

Supplementary Figures S1-3


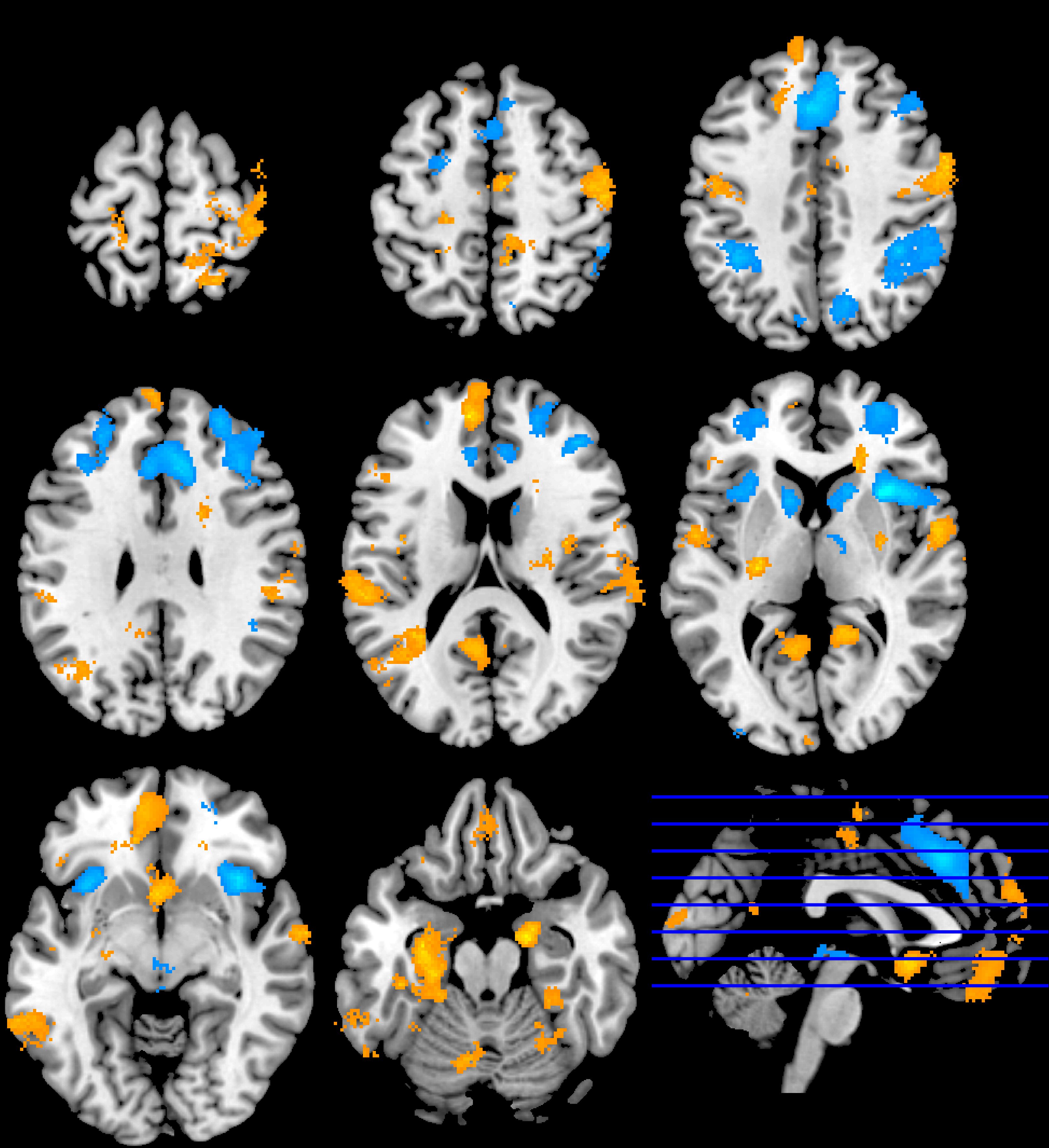


**Figure S1**. RPE effects in adolescents. Whole-brain analysis of RPE effects in the adolescent group displayed at p<.001, uncorrected (cluster extent k=50). Warm colors (yellow) indicate increasing activity with increasing RPEs. Cold colors (blue) indicate increasing activity with decreasing RPEs.


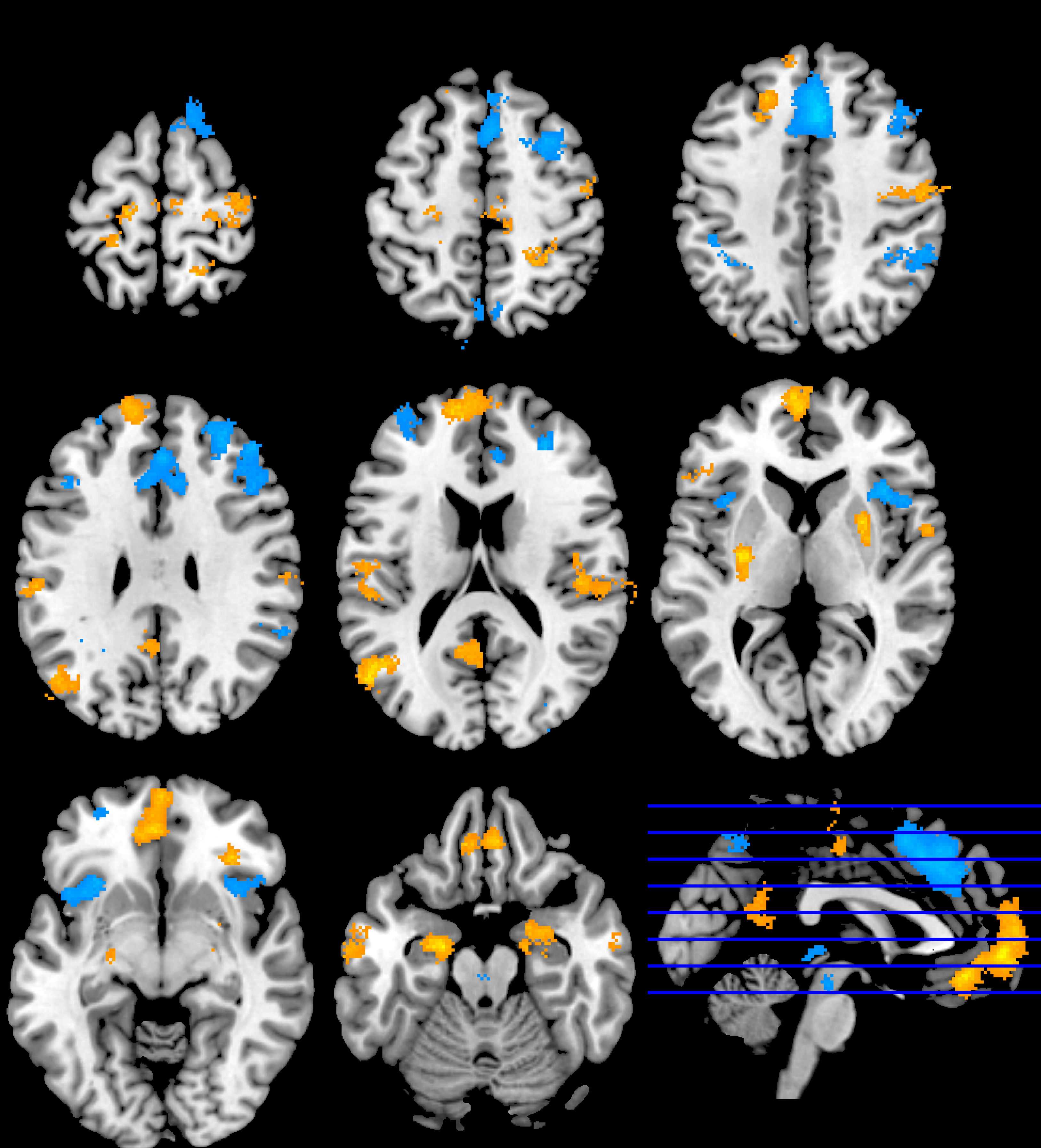


**Figure S2**. RPE effects in adults. Whole-brain analysis of RPE effects in the adult group displayed at p<.001, uncorrected (cluster extent k=50). Warm colors (yellow) indicate increasing activity with increasing RPEs. Cold colors (blue) indicate increasing activity with decreasing RPEs.


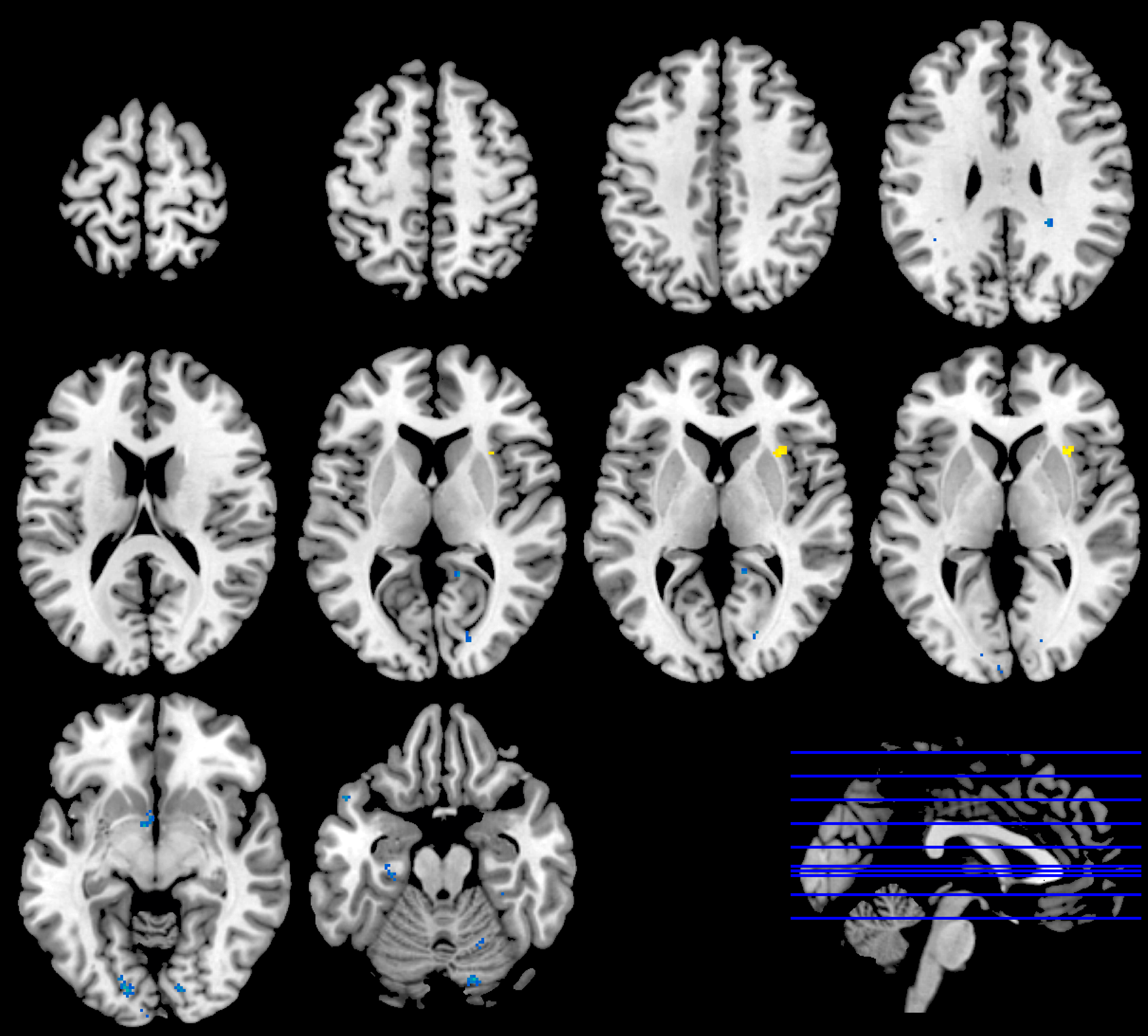


**Figure S3.** Whole-brain differences between adolescents and adults in RPE processing. Unrestricted whole-brain analysis of the 2-sample t-test between adolescents and adults, displayed at p<.001, uncorrected (cluster extent k=10). Warm colors (yellow) show increase activity in adolescents for negative RPEs (cf. Fig. 3B). Cold colors (blue) indicate increased activity for adolescents for positive prediction errors.
